# Racial and Workplace Disparities in Seroprevalence of SARS-CoV-2, Baton Rouge, Louisiana, USA

**DOI:** 10.3201/eid2701.203808

**Published:** 2021-01

**Authors:** Amy K. Feehan, Cruz Velasco, Daniel Fort, Jeffrey H. Burton, Eboni G. Price-Haywood, Peter T. Katzmarzyk, Julia Garcia-Diaz, Leonardo Seoane

**Affiliations:** Ochsner Clinic Foundation, New Orleans, Louisiana, USA (A.K. Feehan, C. Velasco, D. Fort, J.H. Burton, E.G. Price-Haywood, J. Garcia-Diaz, L. Seoane);; The University of Queensland Faculty of Medicine, Ochsner Clinical School, New Orleans (A.K. Feehan, E.G. Price-Haywood, J. Garcia-Diaz, L. Seoane);; Pennington Louisiana State University, Baton Rouge, Louisiana, USA (P.T. Katzmarzyk);; Louisiana State University Health Sciences Center-Shreveport, Shreveport, Louisiana, USA (L. Seoane)

**Keywords:** SARS virus, COVID-19, cross-sectional studies, healthcare disparities, prevalence, seroepidemiologic studies, Baton Rouge, Louisiana, convalescence, coronavirus disease, SARS-CoV-2, severe acute respiratory syndrome coronavirus 2, viruses, respiratory infections, zoonoses, United States

## Abstract

By using paired molecular and antibody testing for severe acute respiratory syndrome coronavirus 2 infection, we determined point prevalence and seroprevalence in Louisiana, USA, during the second phase of reopening. Infections were highly variable by race and ethnicity, work environment, and ZIP code. Census-weighted seroprevalence was 3.6%, and point prevalence was 3.0%.

We previously reported results from a seroprevalence study conducted in New Orleans, Louisiana, USA, which was hit hard early in the coronavirus disease (COVID-19) pandemic (*1*). Baton Rouge is a large metropolitan area roughly 80 miles northwest of New Orleans; at the time of this study, it was in the second phase of reopening after a stay-at-home order. Although the seroprevalence in New Orleans (6.9%) (*1*) was similar to prevalence recorded in Spain (5%), São Paulo, Brazil (4.7%), and New York, USA (6.9%) (*2**,**3*; B.H. Tess, unpub. data, https://doi.org/10.1101/2020.06.29.20142331), Baton Rouge had only 3,427 more cases as of August 2, 2020 (17,093 cases), than New Orleans did by May 16, 2020 (13,666 cases) (*4*). This latest study estimated severe acute respiratory syndrome coronavirus 2 (SARS-CoV-2) infections in the greater Baton Rouge area (Ascension, East Baton Rouge, Livingston, and West Baton Rouge Parishes), with additional information on potential workplace exposures.

The protocol was approved by the Ochsner institutional review board and was designed to enroll and test <2,500 participants at 13 sites throughout Baton Rouge during July 15–31. Recruitment targeted a representative sample by using a method developed by Public Democracy (https://www.publicdemocracy.io) and described elsewhere (*1*,*5*). In contrast to the New Orleans study, in which persons tested were under a stay-at-home order, Baton Rouge was in phase 2 of reopening. A randomized subset of 500,000 Baton Rouge residents were targeted with digital ads for recruitment. Of those, 3,687 volunteers were recruited and restratified according to census designations; 2,309 were invited to participate, 2,179 enrolled and completed testing, and 2,138 were included in our final analysis. A total of 38 persons were excluded because they lived in ineligible ZIP codes, and 3 withdrew consent (Appendix). All study materials were provided in English, Spanish, and Vietnamese. Participants were offered free transportation. Research staff verbally obtained consent from participants and electronically documented consent and survey responses. We then procured blood samples and nasopharyngeal swab specimens from participants.

We used US Food and Drug Administration Emergency Use Authorization–approved tests. Real-time reverse transcription PCR of nasopharyngeal swab specimens was performed by using the Abbott m2000 RealTime system (Abbott, https://www.molecular.abbott). Qualitative IgG blood tests were performed by using the ARCHITECT i2000SR (Abbott). The IgG test meets criteria established by the Centers for Disease Control and Prevention to yield high positive predictive value, which was validated by Ochsner Health laboratory and others (*6**,**7*). Study participants who tested positive on either or both tests were assessed as having been infected with SARS-CoV-2. Point estimates and corresponding 95% CIs for proportions of SARS-CoV-2 exposure (PCR+ or IgG+ tests), point prevalence (PCR+, IgG–), and seroprevalence (IgG+ tests regardless of PCR test result) were estimated for the Baton Rouge area by using raw and census-weighted counts. Unadjusted odds ratios with Firth correction were calculated for all variables.

The sample was 63.6% female and 66.9% white; average age was 48.7 years (range 18–91) and average household size 2.84 persons. The census-weighted estimate of SARS-CoV-2 infections in the sample is 6.6% (6.0%, raw), with 3.0% positive for active viral shedding without detectable antibody, which translates to 16,536 contagious persons. By race and ethnicity, seroprevalence was highest (7.5%) in Black participants, compared with White non-Hispanic (1.8%), Asian non-Hispanic (1.7%), Hispanic of any race (1.6%), and other (2.7%) participants (Table).

**Table:** Prevalence of past and present severe acute respiratory syndrome coronavirus 2 infections by race and ethnicity across Baton Rouge, Louisiana, after phased reopening, July 2020*

Race or ethnicity	Positive no./total no. (% of sample)	Residents >18 y, no. (% of population)*	Any infection, raw,† % (95% CI)	Any infection, weighted,‡ % (95% CI)	Weighted point prevalence,§ % (95% CI)	Weighted seroprevalence,¶ % (95% CI)
Total	128/2138 (100)	551,185 (100)	6.0 (5.0–7.1)	6.6 (5.7–7.7)	3.0 (2.3–3.7)	3.6 (2.8–4.4)
White alone	54/1431 (66.9)	332,445 (60.3)	3.8 (2.9–4.9)	4.2 (3.2–5.2)	2.4 (1.6–3.2)	1.8 (1.1–2.5)
Black or African American alone	57/516 (24.1)	177,950 (32.3)	11.0 (8.5–14.1)	11.0 (8.5–14.1)	3.5 (1.9–5.1)	7.5 (5.2–9.8)
Asian alone	2/59 (3.4)	13,630 (2.5)	3.4 (0.4–11.7)	3.5 (0.0–8.2)	1.7 (0.0–9.1)	1.7 (0.0–9.1)
Other#	1/28 (1.3)	7,025 (1.3)	3.6 (0.1–18.4)	2.7 (0.0–8.7)	0.0	2.7 (0.0–8.7)
Hispanic or Latino, any race	14/104 (4.9)	20,125 (3.7)	13.5 (7.6–21.6)	11.8 (5.6–18.0)	10.1 (4.3–15.9)	1.6 (0.0–4.0)

*Census 2018 population estimates. By August 2, 2020, a total of 17,093 state-aggregated, confirmed cases had been reported in Ascension, East Baton Rouge, Livingston, and West Baton Rouge parishes (*4*).  †Percentage of sample with a PCR-positive test, an IgG-positive test, or both. ‡Census-weighted percentage of PCR-positive test, IgG-positive test, or both calculated to match 2018 racial demographics by parish and combined. §Census-weighted percentage of PCR-positive and IgG-negative tests calculated to match 2018 racial demographics by parish and combined. ¶Census-weighted percentage of IgG–positive tests calculated to match 2018 racial demographics by parish and combined. #Other includes American Indian or Alaska Native, Pacific Islander, and multiracial persons.

The point prevalence and any SARS-CoV-2 infection were mapped by ZIP codes across the greater Baton Rouge area (Appendix). Point prevalence and all infections were highly variable by ZIP code.

Marital status was associated with prevalence (p = 0.0005 by χ^2^ test). Single persons had the highest rate of infection (9.3%), compared with rates for married or cohabitating participants (5.0%), and were 1.9 times more likely to test positive (Figure). Work environment also affected prevalence (p = 0.01 by χ^2^ test); the lowest prevalence was in participants who worked from home part-time and went to a workplace part-time (3.7%). Those who worked primarily outside the home had the highest prevalence (8.2%) and were 2.3 times more likely to test positive than those who worked from home at least part-time. Infection rates varied by occupation (p = 0.01 by χ^2^ test); the lowest positivity was in office workers (3.0%) and increased odds of testing positive occurred in delivery, healthcare, and other public-facing jobs. However, based on seroprevalence, which also varied substantially by occupation (p = 0.03 by χ^2^ test), healthcare workers and public-facing workers bore the brunt of early infections, as demonstrated by higher odds of testing positive for antibodies (Figure).

**Figure:**
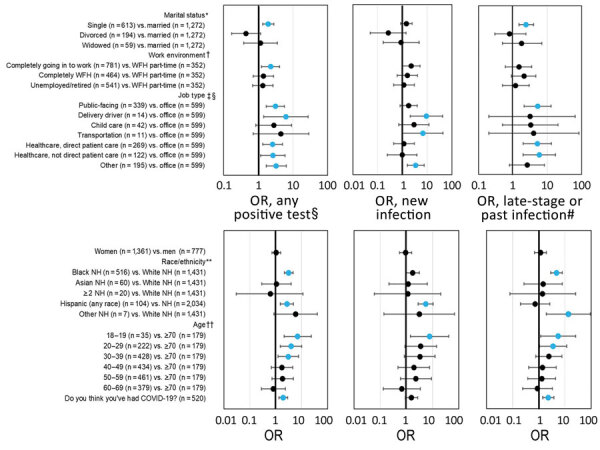
Odds ratios of severe acute respiratory syndrome coronavirus 2 infections by marital status, work environment, and job type after phased reopening in Baton Rouge, Louisiana, USA, July 2020. OR from unweighted logistic regression without covariates with Firth correction are shown with 95% CIs. Reference categories’ percent positivity are married (5.0% any infection, 2.3% seroprevalence), WFH part-time (3.7% any infection, 2.0% seroprevalence), and office workers (3.0% any infection, 1.0% seroprevalence). WFH, work from home; NH, non-Hispanic; OR, odds ratio. *Odds of any infection (p = 0.0005) and seroprevalence (p = 0.03) differ by marital status. †Odds of any infection (p = 0.01) differ by work environment. ‡Odds of any infection (p = 0.01) and seroprevalence (p = 0.03) differ by job type. §Six people did not give an answer for job type; none tested positive on any test. Unemployed/retired people (n = 541) are not included in this category. ¶Percentage and OR of any positive test (PCR+ or IgG+). #Percentage and OR of late-stage or past infections (IgG+, regardless of PCR status). **Odds of any infection (p<0.0001) and seroprevalence (p<0.0001) differ by race and ethnicity. ††Odds of any infection (p<0.0001) and seroprevalence (p = 0.0074) differ by age.

We found the prevalence of SARS-CoV-2 infection in Baton Rouge to be 6.6% but with a heavy concentration of new, contagious infections (3.0%). Persons who were infected early possibly no longer had antibodies. This finding differed from our New Orleans study, which was performed after extensive lockdowns and estimated new infections at 0.9% (*1*). Some populations had higher rates of infection than others, including Black and Hispanic communities and public-facing workers or those who do not work from home.

Appendix Additional information on racial and workplace disparities in seroprevalence of SARS-CoV-2, Baton Rouge, Louisiana, USA.
