# Supplementary material for: Racial and Workplace Disparities in Seroprevalence of SARS-CoV-2, Baton Rouge, Louisiana, USA
Source: Emerg Infect Dis. 2021 Jan;27(1):314–7. doi: 10.3201/eid2701.203808 (PMC7774581; doi:10.3201/eid2701.203808)
Supplement: Appendix — Additional information on racial and workplace disparities in seroprevalence of SARS-CoV-2, Baton Rouge, Louisiana, USA. [file 20-3808-Techapp-s1.pdf]

Article DOI: https://doi.org/10.3201/eid2701.203808

Racial and Workplace Disparities in Seroprevalence of SARS-CoV-2, Baton Rouge, Louisiana, USA

Appendix

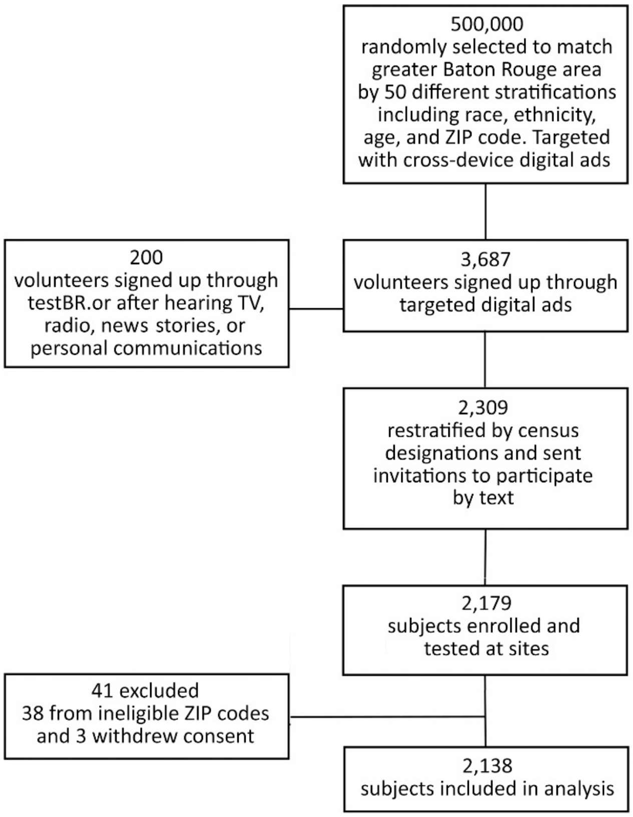

Appendix Figure 1. Flow diagram of recruitment to enrollment and analysis, Baton Rouge, Louisiana, July 2020.

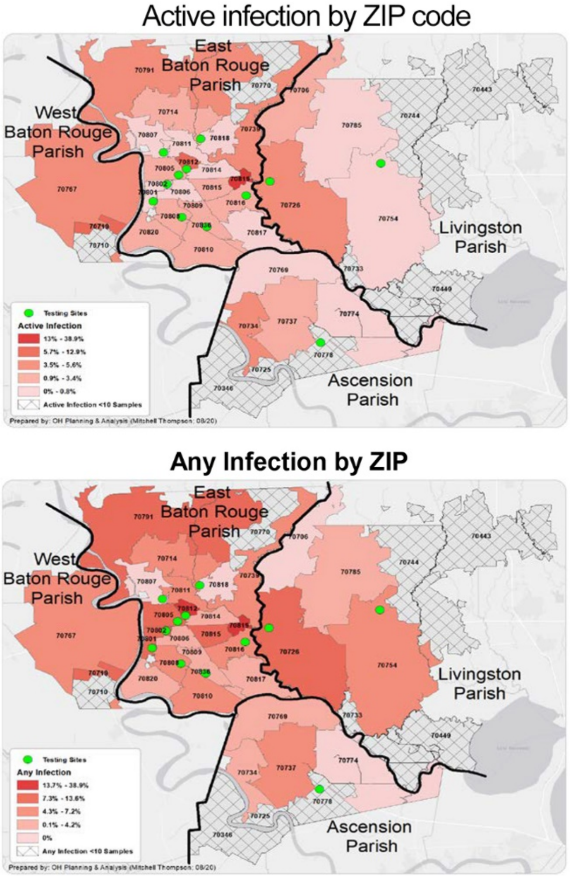

ZIP code	No.	Active infection (%)	Any infection (%)
70808	228	6 (2.6%)	12 (5.3%)
70726	198	11 (5.6%)	18 (9.1%)
70810	178	5 (2.8%)	8 (4.5%)
70816	152	5 (3.3%)	11 (7.2%)
70817	152	1 (0.7%)	4 (2.6%)
70806	146	1 (0.7%)	4 (2.7%)
70769	130	1 (0.8%)	5 (3.8%)
70809	99	2 (2.0%)	4 (4.0%)
70737	89	3 (3.4%)	5 (5.6%)
70815	74	2 (2.7%)	6 (8.1%)
70706	62	2 (3.2%)	0 (0%)
70802	56	0 (0%)	5 (8.9%)
70791	52	2 (3.8%)	4 (7.7%)
70739	50	2 (4.0%)	3 (6.0%)
70820	49	1 (2.0%)	1 (2.0%)
70814	46	0 (0%)	1 (2.2%)
70805	44	1 (2.3%)	6 (13,6%)
70818	40	0 (0%)	0 (0%)
70785	39	0 (0%)	1 (2.6%)
70714	38	1 (2.6%)	2 (5.3%)
70812	31	4 (12.9%)	9 (29.0%)
70734	24	1 (4.2%)	1 (4.2%)
70767	19	1 (5.3%)	1 (5.3%)
70811	18	0 (0%)	1 (5.6%)
70819	18	7 (38.9%)	7 (38.9%)
70754	16	0 (0%)	1 (6.3%)
70807	14	0 (0%)	0 (0%)
70719	11	1 (9.1%)	1 (9.1%)
70774	10	0 (0%)	0 (0%)
70710	8	0 (0%)	0 (0%)
70733	8	1 (12.5%)	1 (12.5%)
70836	7	0 (0%)	1 (14.3%)
70744	5	0 (0%)	0 (0%)
70346	4	0 (0%)	0 (0%)
70725	4	0 (0%)	2 (50.0%)
70770	4	0 (0%)	0 (0%)
70801	4	0 (0%)	0 (0%)
70778	3	0 (0%)	0 (0%)
70821	2	0 (0%)	0 (0%)
70443	1	0 (0%)	0 (0%)
70449	1	0 (0%)	0 (0%)
70707	1	0 (0%)	0 (0%)
70727	1	1 (100%)	1 (100%)

Appendix Figure 2. Heatmaps of coronavirus disease infection in the greater Baton Rouge area, July 2020.
